# Asthma exacerbations and worsenings in patients aged 1–75 years with add-on tiotropium treatment

**DOI:** 10.1038/s41533-020-00193-w

**Published:** 2020-08-31

**Authors:** J. Mark FitzGerald, Eckard Hamelmann, Huib A. M. Kerstjens, Roland Buhl

**Affiliations:** 1 Centre for Heart and Lung Health, Vancouver, BC Canada; 2 Klinik für Kinder- und Jugendmedizin, Evangelisches Klinikum Bethel, Bielefeld, Germany; 3 Allergy Center of the Ruhr University, Bochum, Germany; 4 Department of Pulmonology and Tuberculosis, University Medical Center Groningen, University of Groningen, Groningen, The Netherlands; 5 Groningen Research Institute for Asthma and COPD GRIAC, Groningen, The Netherlands; 6 Pulmonary Department, Johannes Gutenberg University Hospital Mainz, Mainz, Germany

**Keywords:** Asthma, Allergy, Asthma

## Abstract

This review explores the effect of tiotropium Respimat® add-on therapy on asthma exacerbations and worsenings, adverse events (AEs) related to exacerbations and symptoms and any effects on seasonality across the 10 UniTinA-asthma® clinical trials comprising over 6000 patients. When added on to inhaled corticosteroids ± additional therapies, tiotropium significantly reduced the risk of exacerbations and worsenings in adults with symptomatic severe asthma and provided a non-significant improvement in worsenings in adults with symptomatic moderate and mild asthma, which was significant for patients with moderate asthma receiving tiotropium 2.5 µg once daily vs. placebo. Trials in paediatric patients were not powered to assess exacerbations or worsenings, but when AEs related to asthma exacerbations and symptoms were grouped into a composite endpoint and pooled, tiotropium improved outcomes vs. placebo (rate ratio 0.76; 95% confidence interval 0.63, 0.93). The reduction in exacerbations with tiotropium is apparent across all patients during the observed seasonal peaks of these events.

## Introduction

Despite established treatment guidelines for asthma, and reports suggesting that it is possible for most patients to achieve control of their illness, many patients remain symptomatic and have poor disease control^1,2^. Poorly controlled asthma is associated with functional limitations, pulmonary function loss, reduced quality of life and increased risk of exacerbations^1^. Asthma exacerbations represent an acute change in a patient’s usual symptoms and lung function, and are caused by multiple triggers^3^. Symptoms include shortness of breath, coughing, wheezing and chest tightness, each of which can be a frightening experience for the patient^4,5^. The prevention of exacerbations and worsenings, or preventing the future risk of exacerbations and worsenings, is one of the main treatment goals of current asthma guidelines^3,6^. Powering a trial to assess asthma exacerbations requires a sufficient study duration and adequate patient numbers—in particular, those who are unstable and at risk of experiencing exacerbations. Assuming that asthma worsenings are an indicator of asthma exacerbation risk, then asthma worsenings could be used as an alternative study endpoint which has clinical relevance. This could allow for studies of shorter duration and smaller sample sizes^7–9^.

## Defining asthma exacerbations and worsenings

Although definitions have been proposed by groups including the Global Initiative for Asthma (GINA) and the American Thoracic Society/European Respiratory Society (Table 1), there is no clear consensus definition for asthma ‘exacerbations’^10,11^. It is also noted that there is no consensus definition of worsenings, with literature definitions generally including increased use of short-acting β_2_-agonists (SABAs), peak expiratory flow (PEF) changes and increased day- and night-time symptoms^9^. Patients themselves may not understand or be aware of the terms ‘exacerbation’ or ‘worsening’. There has therefore been a suggestion that despite having variable meanings, the use of the terms ‘attack’, ‘episode’ or ‘flare-up’ may facilitate patient and care provider recognition of a deterioration of asthma control and the need to seek additional controller options^3,12^.

**Table 1:** Definitions of asthma exacerbations and worsenings.

Definition	GINA^3^	ATS/ERS^13^	Primo/Mezzo/GraziaTinA-asthma trials in adult patients^19–21^ and Pensie/Ruba/Viva/CanoTinA-asthma in paediatric patients (aged 6–11 years)^14–18^
Asthma exacerbations	Episodes characterised by a progressive increase in symptoms (e.g. shortness of breath, cough, wheezing or chest tightness) and a progressive decrease in lung function that require a change in the patient’s usual treatment	Moderate exacerbations: events that include deterioration in symptoms or lung function, or increased rescue bronchodilator use lasting at least 2 days, that require a temporary change in treatment (with the exception of systemic corticosteroids or hospitalisation) Severe exacerbations: event that requires use of systemic corticosteroids or an increase from the patient’s usual stable maintenance dose for at least 3 days, or hospitalisation	An episode of progressive increase in ≥1 asthma symptom(s) (as compared with usual day-to-day asthma symptoms), or a decline of ≥30% in PEF_a.m._ for ≥2 consecutive days, requiring systemic corticosteroids for ≥3 days
Asthma worsenings			An episode of progressive increase in ≥1 asthma symptom(s) (as compared with usual day-to-day asthma symptoms), or a decline of ≥30% in PEF_a.m._ for ≥2 consecutive days

*ATS* American Thoracic Society, *ERS* European Respiratory Society, *GINA* Global Initiative for Asthma, *PEF*_*a.m.*_ morning peak expiratory flow.

The lack of a single definition for either asthma exacerbations or worsenings makes it difficult to compare outcomes across multiple clinical trials^10,13^. However, this is possible with the UniTinA-asthma® clinical trial database of ten randomised, double-blind, placebo-controlled, parallel-group trials lasting between 12 weeks and 1 year in duration, which all used the same definitions. This allows us to review the effect of tiotropium Respimat® add-on therapy on episodes of asthma exacerbations and worsenings, as well as adverse events (AEs) related to exacerbations and symptoms, over a large patient population.

Tiotropium Respimat® has been shown to be an efficacious and well-tolerated add-on treatment to inhaled corticosteroid (ICS) therapy with or without additional controllers, with comparable safety and efficacy to long-acting β_2_-agonists (LABAs)^14–22^. A systematic review and meta-analysis comparing the efficacy and safety of tiotropium with that of LABAs as add-on to ICS in patients with uncontrolled persistent asthma aged 12 years and older reported comparable improvements in clinical outcomes^22^. Similarly, in paediatric patients aged 4–17 years with asthma, a recent literature review of LABAs, leukotriene receptor antagonists (LTRAs) and tiotropium reported that tiotropium and LABAs have similar efficacy and provide greater improvements in lung function than LTRAs as add-on to ICS. All three add-on therapies had comparable safety profiles^23^.

Here, we collate and review the published literature on the effect of add-on tiotropium treatment on asthma exacerbations and worsenings in patients aged 1–75 years. We also look at any effect of tiotropium on seasonality of asthma exacerbations and worsenings^14–21^ (Supplementary Figure 1).

## Treatment for asthma exacerbations and worsenings

**Fig. 1:**
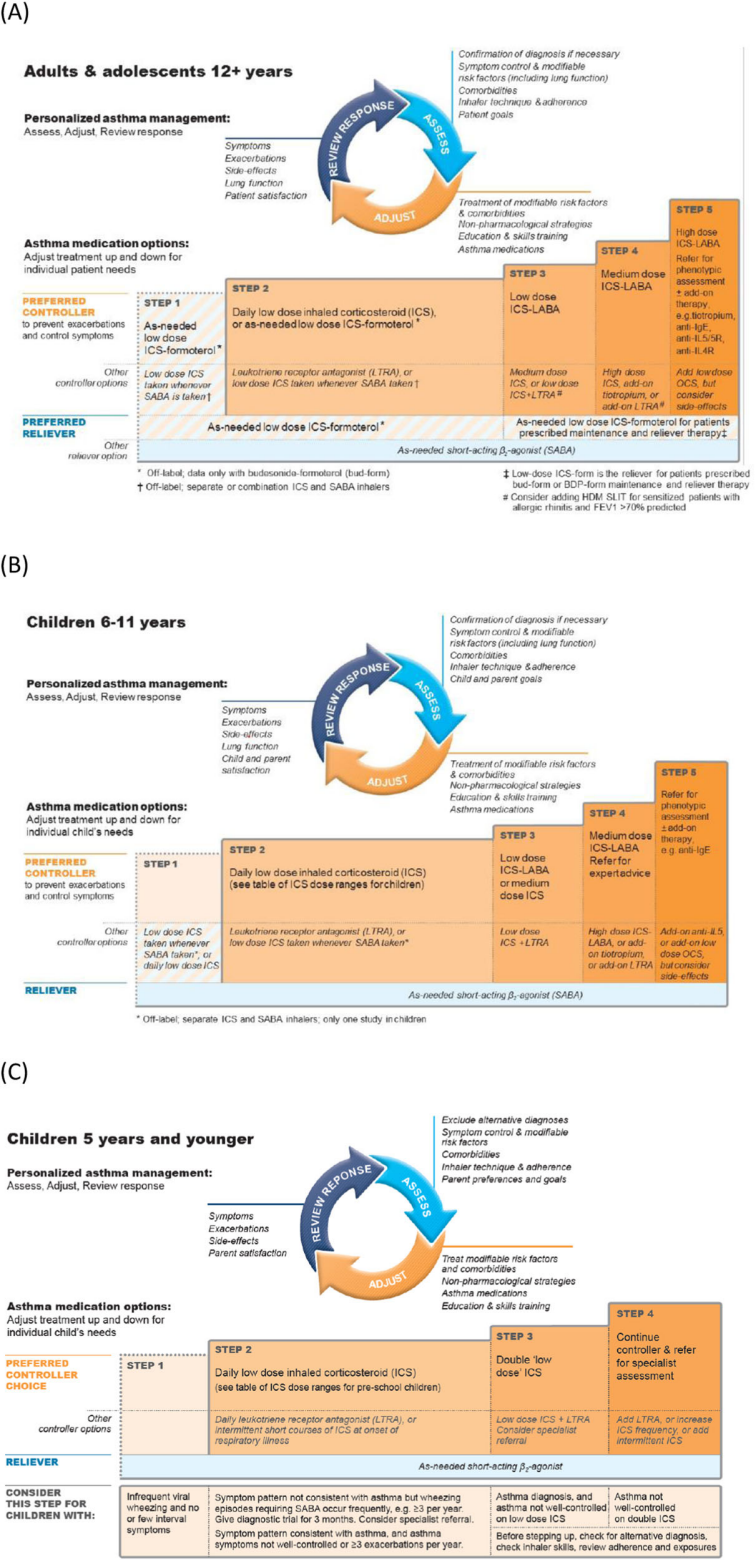
GINA treatment recommendations for patients aged ≥12 years, 6–11 years and ≤5 years^3^. GINA treatment recommendations for stepping up or stepping down of asthma controller and reliever therapy for the management of asthma for patients aged (**a**) ≥12 years, (**b**) 6–11 years and (**c**) ≤5 years. © 2020, Global Initiative for Asthma, reproduced with permission. FEV_1_ forced expiratory volume in 1s, GINA Global Initiative for Asthma, HDM house dust mite, ICS inhaled corticosteroid, Ig immunoglobulin, IL interleukin, LABA long-acting β_2_-agonist, LTRA leukotriene receptor antagonist, OCS oral corticosteroids, SABA short-acting β_2_-agonist, SLIT sublingual immunotherapy.

GINA recommends that controller medication is adjusted in a stepwise approach—stepped up if control is not achieved and stepped down once good asthma control has been achieved for at least 3 months—in order to find the lowest appropriate treatment for the patient that controls both symptoms and exacerbations (Fig. 1)^3,24^.

The long-acting muscarinic antagonist tiotropium Respimat® is indicated in the EU as add-on maintenance bronchodilator treatment in patients aged 6 years and older with severe asthma who experienced one or more severe asthma exacerbation in the preceding year^25^. In the United States, it is indicated for the long-term, once-daily maintenance treatment of asthma in patients aged 6 years and older^26^. It has been evaluated as add-on to at least ICS therapy in >6000 patients aged 6–75 years with symptomatic asthma, and aged 1–5 years with persistent asthmatic symptoms, in the comprehensive Phase II and III UniTinA-asthma® clinical trial programme. Within the individual trials, it has been shown to be an efficacious and well-tolerated add-on treatment to ICS therapy with or without additional controllers^14–21^.

## Exacerbations as an endpoint in paediatric studies

Paediatric patients are a vulnerable population, with exacerbations being a particular concern. However, powering a placebo-controlled trial to assess asthma exacerbations as a primary endpoint in paediatric patients can present ethical considerations^27^. If exacerbations are anticipated, not providing the best proven standard of care exposes paediatric patients to unnecessary risk^27,28^. The trial length required to gather sufficient exacerbations data is also problematic for children, especially for those receiving a placebo. Paediatric patients receiving a placebo may be more likely to withdraw from a trial than those receiving the active treatment due to experiencing exacerbations^28^. Furthermore, for many patients, the effect of a medication on exacerbations may be a lower priority than its impact on more frequently reported outcomes, such as asthma symptoms or lung function. Subsequently, alternative endpoints may be preferable when assessing the efficacy of asthma treatments in terms of exacerbation risk reduction. Szefler et al.^29^ previously demonstrated that using AEs related to exacerbations and symptoms as endpoints was aligned to using exacerbations as an endpoint, and that it was possible to detect treatment differences with AE reporting when exacerbation data were not available.

## Seasonality of asthma exacerbations and worsenings

Although asthma exacerbations and worsenings may present sporadically, they are often determined by seasons^30,31^. Peaks may mirror patterns of allergen exposure and prevalence of respiratory viral infections^30,31^.

As in adults, AEs related to asthma exacerbations and symptoms in paediatric patients also display seasonal patterns. However, these patterns differ from those seen in adults, with paediatric patients more likely to experience exacerbations or worsenings in the spring and autumn months^32,33^. The autumn peak of exacerbations in children and adolescents is largely attributed to an increased frequency of rhinovirus infections among children returning to school following the summer break^34,35^.

## Studies included within review

Tiotropium Respimat® has been evaluated as add-on to at least ICS therapy in patients aged 6–75 years with symptomatic asthma and 1–5 years with persistent asthmatic symptoms in the comprehensive Phase II and III UniTinA-asthma clinical trial programme that included >6000 patients (Table 2). We collated previously published data from the ten UniTinA-asthma clinical trials, to assess and discuss the effect of tiotropium Respimat® add-on therapy on asthma exacerbations and worsenings, as well as AEs related to exacerbations and symptoms, in patients aged 1–75 years. The clinical trial programme included two replicate trials in adults with symptomatic severe asthma (PrimoTinA-asthma [NCT00772538/NCT00776984])^19^, two replicate trials in adults with symptomatic moderate asthma (MezzoTinA-asthma [NCT01172808/NCT01172821])^20^ and one trial in adults with symptomatic mild asthma (GraziaTinA-asthma [NCT01316380])^21^. In adolescents (aged 12–17 years), there were two trials: one in patients with symptomatic severe (PensieTinA-asthma [NCT01277523])^15^ and one in patients with symptomatic moderate asthma (RubaTinA-asthma [NCT01257230])^14^. Similarly, there were two trials in children (aged 6–11 years): one in patients with symptomatic severe (VivaTinA-asthma [NCT01634152])^18^ and one in patients with symptomatic moderate asthma (CanoTinA-asthma [NCT01634139])^17^. In children aged 1–5 years, there was one trial in patients with persistent asthmatic symptoms (NinoTinA-asthma [NCT01634113])^16^.

**Table 2:** Study designs.

Study name (ClinicalTrials.gov number)	Asthma severity	Tiotropium Respimat dose	Patients (*N*)^a^	Study duration (weeks)	Patient age (years)^b^	History of asthma^b^	ACQ mean score^b^	Lung function (FEV_1_ % predicted)^b,c,d^
PrimoTinA-asthma (NCT00772538)^19^	Severe	Tiotropium (5 µg QD) vs. placebo + LABA as add-on to ICS (800 µg budesonide/equivalent)	459	48	18–75	≥5 years	≥1.5	≤80%
PrimoTinA-asthma (NCT00776984)^19^	Severe	Tiotropium (5 µg QD) vs. placebo + LABA as add-on to ICS (800 µg budesonide/equivalent)	453	48	18–75	≥5 years	≥1.5	≤80%
MezzoTinA-asthma (NCT01172808)^20^	Moderate	Tiotropium (5 or 2.5 µg QD) vs. LABA vs. placebo as add-on to ICS (400–800 µg budesonide/equivalent)	1071	24	18–75	3 months	≥1.5	60–90%
MezzoTinA-asthma (NCT01172821)^20^	Moderate	Tiotropium (5 or 2.5 µg QD) vs. LABA vs. placebo as add-on to ICS (400–800 µg budesonide/equivalent)	1032	24	18–75	3 months	≥1.5	60–90%
GraziaTinA-asthma (NCT01316380)^21^	Mild	Tiotropium (5 or 2.5 µg QD) vs. placebo as add-on to ICS (200–400 µg budesonide/equivalent)	465	12	18–75	3 months	≥1.5	60–90%
PensieTinA-asthma (NCT01277523)^15^	Severe	Tiotropium (5 or 2.5 µg QD) vs. placebo + ≥1 controller therapy as add-on to high-dose ICS > 400 µg budesonide/equivalent in patients aged 12–14 years and 800–1600 µg budesonide/equivalent in patients aged 15–17 years) or ≥2 controller therapies as add-on to medium-dose ICS (200–400 µg budesonide/equivalent in patients aged 12–14 years and 400–800 µg budesonide/equivalent in patients aged 15–17 years)	392	12	12–17	≥3 months	≥1.5	60–90%
RubaTinA-asthma (NCT01257230)^14^	Moderate	Tiotropium (5 or 2.5 µg QD) vs. placebo ± LTRA as add-on to ICS (200–800 µg budesonide/equivalent in patients aged 12–14 years and 400–800 µg budesonide/equivalent in patients aged 15–17 years)	398	48	12–17	≥3 months	≥1.5	60–90%
VivaTinA-asthma (NCT01634152)^18^	Severe	Tiotropium (5 or 2.5 µg QD) vs. placebo + ≥1 controller therapy as add-on to high-dose ICS (>400 µg budesonide/equivalent) or ≥2 controller therapies as add-on to medium-dose ICS (200–400 µg budesonide/equivalent)	401	12	6–11	≥6 months	≥1.5	60–90%
CanoTinA-asthma (NCT01634139)^17^	Moderate	Tiotropium (5 or 2.5 µg QD) vs. placebo ± LTRA as add-on to ICS (200–400 µg budesonide/equivalent)	403	48	6–11	≥6 months	≥1.5	60–90%
NinoTinA-asthma (NCT01634113)^16^	Persistent asthmatic symptoms	Tiotropium (5 µg or 2.5 µg QD) vs. placebo ± additional maintenance therapies as add-on to ICS (dose not reported)	102	12	1–5	≥6-month history of persistent asthmatic symptoms	Not reported	≤90%^e^

*ACQ* Asthma Control Questionnaire, *FEV*_*1*_ forced expiratory volume in 1 s, *ICS* inhaled corticosteroids, *LABA* long-acting β_2_-agonist, *LTRA* leukotriene receptor antagonist, *QD* once daily.

^a^Number of patients randomised to receive treatment with either tiotropium or placebo.

^b^Inclusion criteria.

^c^Post-bronchodilator.

^d^At screening.

^e^In children aged 5 years who were capable of doing technically acceptable lung function tests.

The two replicate PrimoTinA-asthma trials in adults with symptomatic severe asthma, when pooled by the study investigators, were the only trials powered to assess exacerbations, with a primary predefined efficacy endpoint of time to first asthma exacerbation^19^. Rates of asthma exacerbations were secondary endpoints in the MezzoTinA-, GraziaTinA-, PensieTinA-, RubaTinA-, VivaTinA- and CanoTinA-asthma trials in adults, adolescents and children aged 6–11 years with symptomatic asthma. As such, the individual trials were not powered to assess asthma exacerbations, given the anticipated and ultimately small proportion of patients who experienced exacerbations^14,17,18,36–38^.

## Defining asthma exacerbations and worsenings

Across the five trials in adults with symptomatic asthma and four trials in paediatric patients aged 6–17 years, ‘asthma worsening’ was defined as an episode of progressive increase in ≥1 asthma symptom(s) (as compared with usual day-to-day asthma symptoms), or a decline of ≥30% in morning PEF (PEF_a.m._) for ≥2 consecutive days^14,17,18,36–38^. ‘Asthma exacerbation’ was defined as an episode of asthma worsening requiring systemic corticosteroids for ≥3 days (Table 1)^14,17,18,36–38^. The number of patients with asthma worsenings includes all patients who had asthma exacerbations.

AEs related to exacerbations and symptoms were used as an alternative endpoint in the four clinical trials in paediatric patients aged 6–17 years and the one trial in patients aged 1–5 years (NinoTinA-asthma). AEs were recorded among safety assessments in the UniTinA-asthma clinical trials and assigned to specific MedDRA 18.1 preferred terms^39,40^. These preferred terms have previously been described^41^. Those related to asthma exacerbations and asthma-related symptoms were grouped into an alternative efficacy endpoint, thus giving an indication of worsening of disease or exacerbations^36,37,39,40,42–47^. Szefler et al.^29^ previously demonstrated that using AEs related to exacerbations and symptoms as endpoints was aligned to using exacerbations as an endpoint, and that it was possible to detect treatment differences with AE reporting when exacerbation data were not available.

## Exacerbations in adults with symptomatic severe asthma

**Fig. 2:**
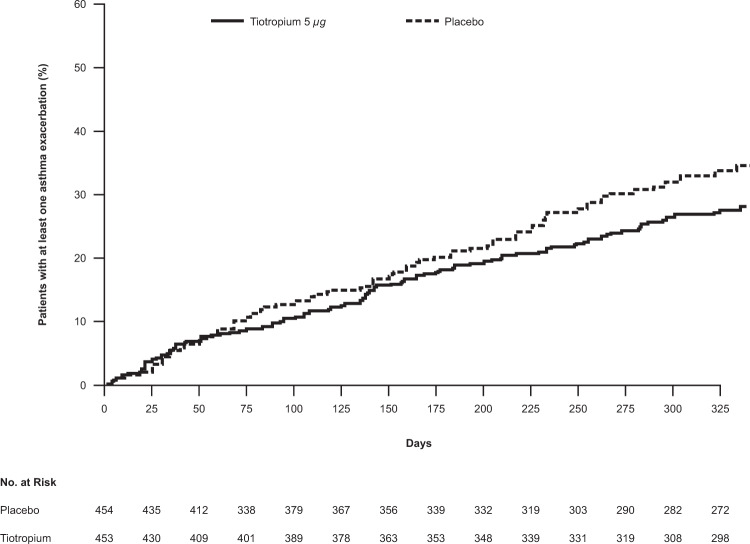
Time to first asthma exacerbation (PrimoTinA-asthma)^19^. A Kaplan–Meier curve of adults with symptomatic severe asthma receiving either tiotropium 5 µg once daily or placebo who reported at least one asthma exacerbation over the 48-week trial period. From ref. Kerstjens et al.^19^. Copyright © 2012 Massachusetts Medical Society. Reprinted with permission.

Tiotropium was shown to significantly reduce the risk of experiencing at least one asthma exacerbation in adults with symptomatic severe asthma compared with placebo (hazard ratio [HR] 0.79; 95% confidence interval [CI] 0.62, 1.00; *P* = 0.0343) (Fig. 2)^19,42^.

## Worsenings in adults with asthma across severities

**Fig. 3:**
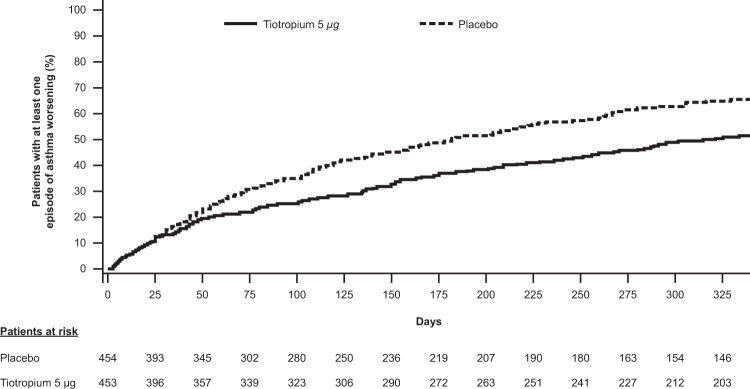
Time to first episode of asthma worsening (PrimoTinA-asthma)^19^. A Kaplan–Meier curve of adults with symptomatic severe asthma receiving either tiotropium 5 µg once daily or placebo who reported at least one episode of asthma worsening over the 48-week trial period. From ref. ^19^. Copyright © 2012 Massachusetts Medical Society. Reprinted with permission.

**Fig. 4:**
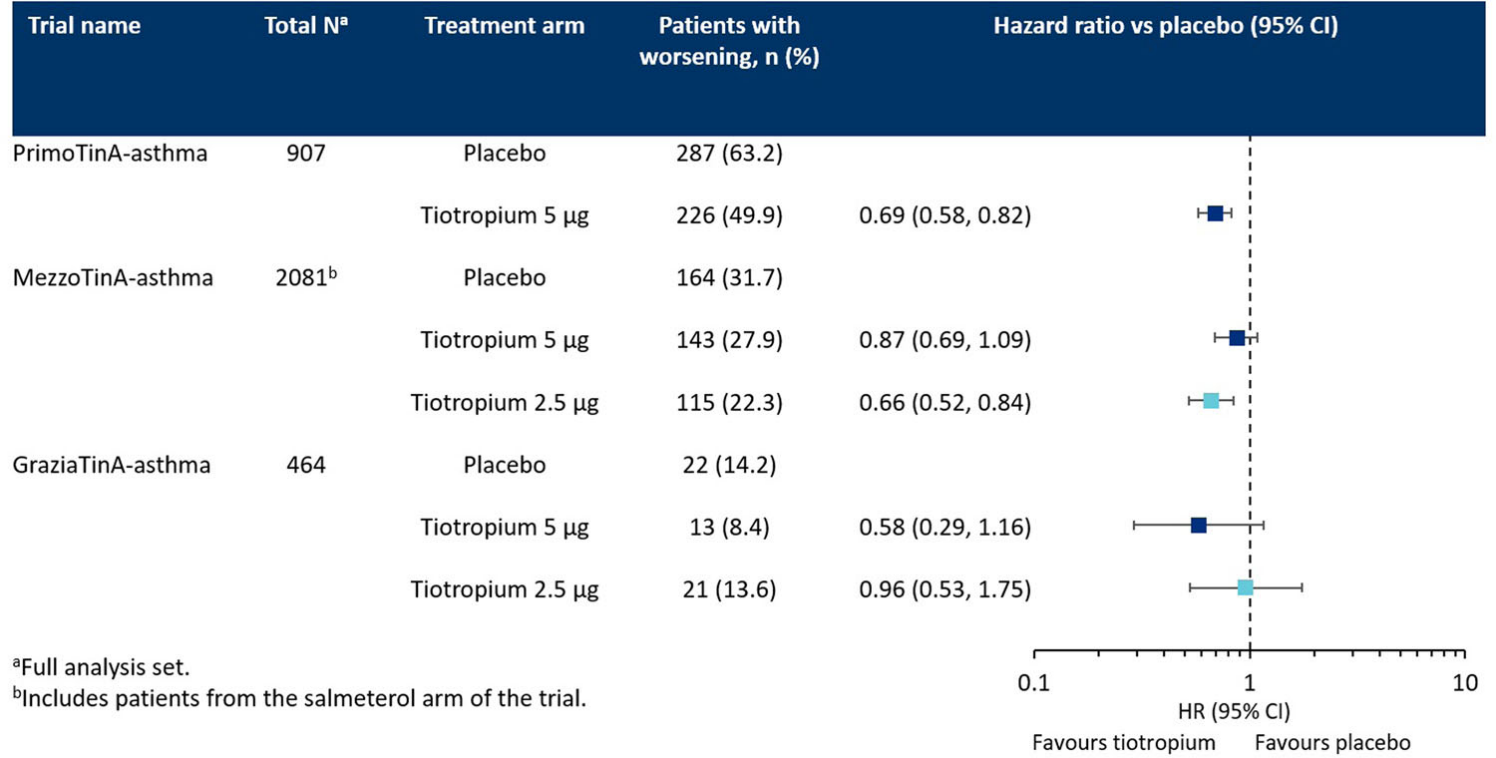
Improvements in time to first episode of asthma worsening in patients in the PrimoTinA-, MezzoTinA- and GraziaTinA-asthma trials receiving tiotropium add-on therapy compared with placebo. Comparison of number of adults with asthma receiving either tiotropium (5 or 2.5 µg) or placebo once-daily reporting at least one episode of asthma worsening. The vertical axis represents an equal risk of experiencing an episode of asthma worsening when receiving either placebo or tiotropium. HR values <1 demonstrate that tiotropium provides a reduction in risk compared with placebo. An upper CI of <1 demonstrates that the reduction in risk is statistically significant. CI confidence interval, HR hazard ratio.

When pooled, the PrimoTinA-asthma studies were sufficiently powered to assess the secondary endpoint of time to first asthma worsening. Tiotropium 5 µg per day was shown to significantly increase the time to first episode of asthma worsening compared with placebo (HR 0.69; 95% CI 0.58, 0.82; *P* < 0.001) (Figs 3, 4)^19^. The time to first episode of asthma worsening was also a secondary endpoint in the two pooled MezzoTinA-asthma trials and the GraziaTinA-asthma trial (Fig. 4). Tiotropium 5 µg showed no significant reduction in the risk of asthma worsenings in patients with symptomatic moderate or mild asthma compared with placebo (HR 0.87; 95% CI 0.69, 1.09 and HR 0.58; 95% CI 0.29, 1.16, respectively). Tiotropium 2.5 µg per day, however, provided a significant reduction in the risk of asthma worsenings in patients with symptomatic moderate asthma in the MezzoTinA-asthma trials (HR 0.66; 95% CI 0.52, 0.84), but no significant reduction in patients with symptomatic mild asthma in the GraziaTinA-asthma trial (HR 0.96; 95% CI 0.53, 1.75) compared with placebo^20,36^. The MezzoTinA-asthma study authors did not elaborate on potential explanations for why tiotropium 2.5 µg provided a reduction in the risk of asthma worsenings, but not tiotropium 5 µg. It is worth noting that for the MezzoTinA- and GraziaTinA-asthma trials, investigators were unable to calculate the time to first asthma worsening, as <50% of patients in each treatment group had one or more incidence of asthma worsening. Therefore, there is a need for larger trials of longer duration to fully assess the potential effects of tiotropium on asthma worsenings.

In a systematic review and meta-analysis, Sobieraj et al.^22^ compared the effect of tiotropium add-on therapy in reducing risk of asthma worsenings with that of LABA add-on therapy. The authors pooled data from the adult and adolescent trials in severe asthma (PrimoTinA and PensieTinA-asthma) and moderate and mild asthma (MezzoTinA-, GraziaTinA- and RubaTinA-asthma). When pooled, the data suggest that tiotropium significantly reduces the risk of asthma worsenings when added to ICS alone (MezzoTinA-, GraziaTinA-, and RubaTinA-asthma: risk ratio, 0.81, 95% CI 0.68, 0.97) and when added to ICS and additional controller therapies (PrimoTinA- and PensieTinA-asthma: risk ratio 0.78, 95% CI 0.72, 0.86). The reduction in risk was reported to be comparable to that provided by LABA as add-on to ICS.

## Seasonality of asthma exacerbations and worsenings in adults

**Fig. 5:**
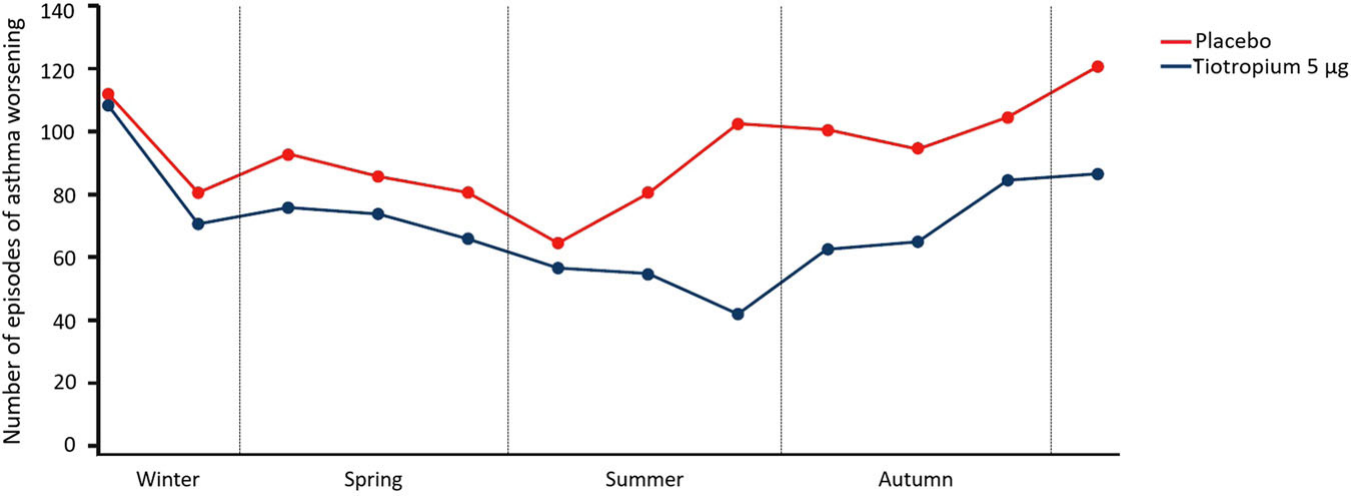
Number of episodes of asthma worsening by season in patients within the two PrimoTinA-asthma trials^48^. Number of adult patients with symptomatic severe asthma reporting at least one episode of asthma worsening receiving either tiotropium 5 µg once daily or placebo plotted by month. Data from the Southern Hemisphere were shifted by 6 months (July = Month 1, January = Month 7) to align the seasonal time periods based on meteorological definitions. Patients were recruited and commenced the 48-week treatment period across all months of the year. From ref. ^48^. Copyright © 2020. Reprinted with permission.

Data from the placebo group of the two PrimoTinA-asthma trials have provided further evidence of the autumn and winter peaks of asthma worsenings in adults with symptomatic severe asthma. Although the exact cause of the peaks was not specified (e.g. if they were attributable to allergens or viral infections), the PrimoTinA-asthma trials have demonstrated that, when episodes were plotted by month, tiotropium 5 µg add-on treatment reduced the number of episodes of asthma worsenings in adults with symptomatic severe asthma compared with placebo across all seasons, but especially during the autumn peak (Fig. 5)^48^. Patients were recruited and commenced the 48-week treatment period across all months of the year, so this observation was not attributable to duration of treatment^19^.

## Worsenings in paediatric patients aged 6–17 years

**Fig. 6:**
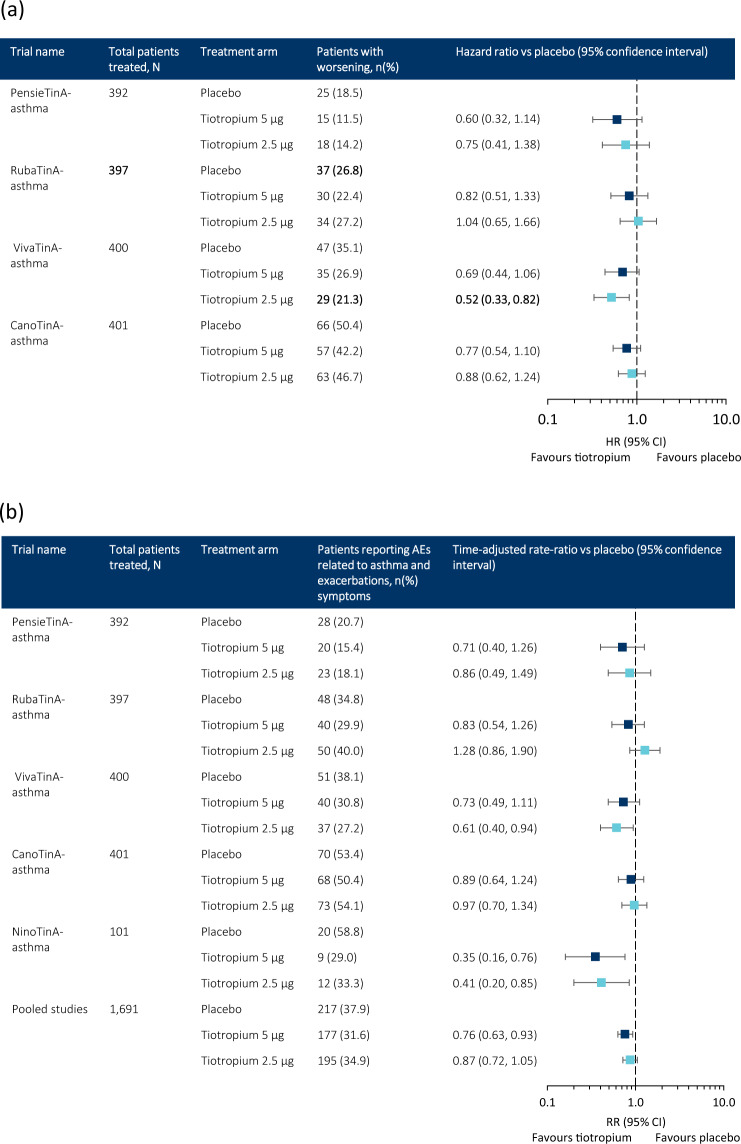
Paediatric patients reporting asthma worsenings and adverse events related to asthma exacerbations or symptoms^29^. Comparison of number of patients aged 1–17 years with asthma or persistent asthmatic symptoms receiving either tiotropium (5 or 2.5µg) or placebo once daily reporting (**a**) asthma worsenings or (**b**) AEs related to asthma exacerbations or symptoms. The vertical axis represents an equal risk of experiencing an AE related to asthma exacerbations or symptoms when receiving either placebo or tiotropium. HR/RR values <1 demonstrate that tiotropium provides a reduction in risk compared with placebo. An upper CI of <1 demonstrates that the reduction in risk is statistically significant. AE adverse event, CI confidence interval, HR hazard ratio, RR rate ratio. Figure **b** is from ref. ^29^. Copyright © 2018. Reprinted with permission.

Tiotropium 5 µg per day generally reduced the risk of asthma worsening in paediatric patients aged 6–17 years with symptomatic moderate or mild asthma (HRs 0.60–0.82), although changes were not statistically significant (Fig. 6a)^29^. Tiotropium 2.5 µg per day also provided numerical reductions in the risk of asthma worsenings in patients aged 6–17 years in the PensieTinA- and CanoTinA-asthma trials, and statistically significant improvements in the VivaTinA-asthma trial^29^. As with the trials in adults with symptomatic moderate and mild asthma, <50% of patients in each treatment group had one or more incidence of asthma worsening^29^, therefore suggesting the need for larger trials of longer durations. These results are in line with those reported by Vogelberg et al.^23^ in a recent literature review of three add-on therapies including tiotropium, where the authors reported significant variability in the design of LABA and LTRA studies in paediatric patients aged 4–17 years, and noted no difference in the risk of exacerbations requiring oral corticosteroids (OCS) between LABAs and LTRA plus ICS compared with ICS alone. The authors reported that tiotropium provided improvements in time to first exacerbation requiring OCS when added to ICS vs. placebo.

## AEs related to paediatric asthma exacerbations and symptoms

Using a composite endpoint in the five UniTinA-asthma clinical trials in paediatric patients aged 1–17 years, which has been demonstrated to be aligned to using exacerbations as an endpoint^29^, there was a reduction in the number of paediatric patients reporting AEs related to asthma exacerbations and symptoms following treatment with tiotropium 5 µg compared with placebo. This reduction was significant when data were pooled (rate ratio 0.76; 95% CI 0.63, 0.93) (Fig. 6b)^29^. Although pooling of the data allows for a greater number of patients to be analysed, thereby providing greater power, it should be noted that the pooled analysis includes children of different age groups and asthma severities, and therefore not all data may have been comparable. When making clinical decisions, data from the appropriate age group and asthma severity should also be considered.

## Seasonality in paediatric patients with asthma

**Fig. 7:**
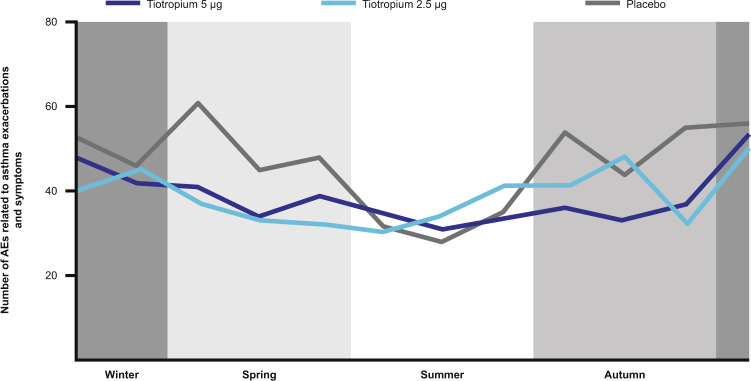
Seasonality of AEs related to asthma exacerbations and symptoms in paediatric patients within the PensieTinA-, RubaTinA-, VivaTinA-, CanoTinA- and NinoTinA-asthma trials^41^. Number of patients aged 1–17 years with asthma or persistent asthmatic symptoms receiving either tiotropium (5 or 2.5 µg) or placebo once daily reporting AEs related to asthma exacerbations and symptoms plotted by month. Data from the Southern Hemisphere were shifted by 6 months (July = Month 1, January = Month 7) to align the seasonal time periods based on meteorological definitions. Patients were recruited and commenced the trial treatment periods across all months of the year. AE adverse event. From ref. ^41^. Copyright © 2019. Reprinted with permission.

Pooled data from the placebo groups of the PensieTinA-, RubaTinA-, VivaTinA-, CanoTinA- and NinoTinA-asthma trials demonstrate seasonal peaks in AEs related to exacerbations and symptoms during the spring and autumn months. As with the PrimoTinA-asthma trials, the causes of these peaks are not specified, but data from the tiotropium add-on therapy groups demonstrate that both tiotropium 5 and 2.5 µg reduced these peaks in paediatric patients and could, therefore, represent an additional intervention for the prevention of seasonal and predictable peaks in AEs relating to asthma exacerbations and symptoms (Fig. 7)^40,41^.

## Conclusions

The definitions of asthma exacerbations and asthma worsenings often vary across different trials, making the comparison of outcomes across multiple clinical trials and studies difficult. By using common definitions throughout the UniTinA-asthma clinical trial programme, it was demonstrated that tiotropium 5 µg is effective in reducing asthma exacerbations and worsenings in adult patients with symptomatic severe asthma. In adults with mild to moderate-severe asthma, tiotropium 5 µg provided a non-significant improvement in the time to first episode of asthma worsening compared with placebo, and in symptomatic moderate asthma, tiotropium 2.5 µg provided a significant improvement. In the five UniTinA-asthma trials in paediatric patients, which analysed exacerbations as a safety endpoint, tiotropium provided significant reductions in the number of patients aged 1–5 years with persistent asthmatic symptoms reporting AEs relating to asthma exacerbations and symptoms compared with placebo. Evidence was limited for the clinical benefit of tiotropium on asthma worsenings or improvements in the number of patients aged 6–17 years reporting AEs relating to asthma exacerbations and symptoms compared with placebo. The reduction in exacerbations with tiotropium was apparent across all patients during the observed seasonal peaks of these events.

### Reporting summary

Further information on experimental design is available in the Nature Research Reporting Summary linked to this paper.

## Supplementary information

Reporting Summary

Supplementary Figure 1
