# Supplementary material for: Asthma exacerbations and worsenings in patients aged 1–75 years with add-on tiotropium treatment
Source: NPJ Prim Care Respir Med. 2020 Aug 31;30:38. doi: 10.1038/s41533-020-00193-w (PMC7459309; doi:10.1038/s41533-020-00193-w)

Supplementary Figure 1. Graphical abstract

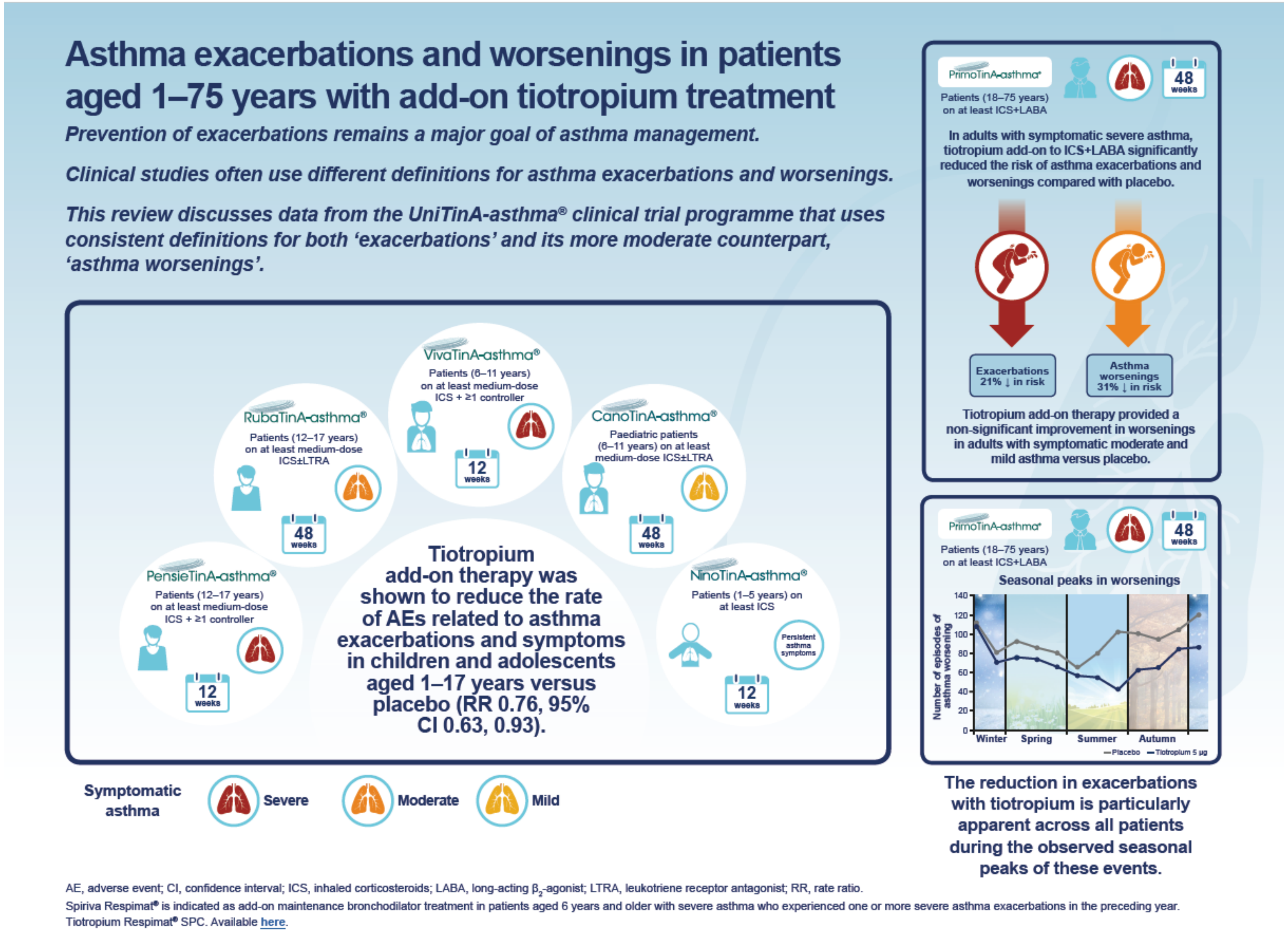

Supplement: Supplementary file 2 — Supplementary Figure 1 [file 41533_2020_193_MOESM2_ESM.pdf]
